# Physiological Changes in Pregnant Women Due to Hormonal Changes

**DOI:** 10.7759/cureus.55544

**Published:** 2024-03-05

**Authors:** Sohan B Jee, Anupama Sawal

**Affiliations:** 1 Medicine, Jawaharlal Nehru Medical College, Datta Meghe Institute of Higher Education & Research, Sawangi (Meghe) Wardha, Wardha, IND; 2 Anatomy, Jawaharlal Nehru Medical College, Datta Meghe Institute of Higher Education & Research, Sawangi (Meghe) Wardha, Wardha, IND

**Keywords:** gastrointestinal, endocrinology, cardiovascular effects, hormonal changes, physiological changes, pregnancy

## Abstract

Pregnancy affects many organ systems and causes significant physiological changes that are mainly caused by changes in hormone levels. This review explores the complex interactions between pregnancy-related hormonal changes and renal function, providing insights into the practical applications of these relationships. Extensive literature searches were conducted, combining data from several sources to produce thorough knowledge. Essential discoveries include changes in renal hemodynamics, calcium/phosphorus level variations, thyroid gland hypertrophy, changed function, and cardiovascular adaptations. The review also addresses how sex hormones affect immunological responses, emphasizing their importance for conditions like multiple sclerosis. Additionally impacted is the gastrointestinal tract, which results in symptoms like nausea and heartburn. Comprehending these physiological changes is essential for proficient therapeutic handling, guaranteeing the best possible health for both the mother and the fetus. The study emphasizes the importance of specialized medical treatment during pregnancy and calls for more investigation to clarify the intricacies of these physiological changes.

## Introduction and background

The substantial physiological changes that occur in various organ systems, beginning at conception and continuing during pregnancy, may impact research into endocrine disorders [1]. A sensitive negative feedback loop regulates thyroid hormone. The anterior pituitary secretes thyroid-stimulating hormone (TSH), which is regulated by thyrotropin-releasing hormone, which, in turn, regulates the thyroid’s release of thyroid hormones (triiodothyronine (T3) and thyroxine (T4)). Thyroid hormone affects metabolism and the functions of many cells and organs. This critical role is reflected in thyroid dysfunction’s apparent signs and symptoms [2]. Hormonal or genetic variables are thought to influence disease progression, and sex hormones such as estrogen, progesterone, prolactin, and androgens likely play a role in the complex mechanisms underlying diseases such as multiple sclerosis (MS). Numerous hormone-related physiological situations in women, such as puberty, pregnancy, and childbirth, have a significant impact on the prevalence and progression of disease and menopause [3].

The investigation into physiologic and functional variations between the sexes is making progress. In addition to circadian rhythms, cancer therapies, cardiovascular disorders, neuropathology, and many other conditions, we are constantly discovering more about how sex and the hormones that are released during it affect these conditions. Because sex hormones affect so many physiological systems and organs, there are typically changes in physiological function between the various menstrual cycle phases. Nearly all physiological systems undergo significant changes during pregnancy [4]. Numerous hormonal and physiological changes occur during different stages of pregnancy, which contribute to acne development even though the pathogenesis is complex and poorly understood. Pregnancy acne is frequently inflammatory, affects the trunk, and is at its worst in the second and third trimesters [5].

Pregnancy affects almost every component of kidney function. A feat of biology is the synchronization of the changes. Renal and systemic hemodynamics are denoted by vasodilation and significant volume expansion. Elevations of 80% and 50% in the renal plasma flow (RPF) and glomerular filtration rate (GFR) are noted compared to pre-pregnancy values. A slight rise in proteinuria and glucosuria, a fall in serum osmolality, and a decrease in blood sodium levels result from modest alterations in tubular activity and the processing of water and electrolytes. Pregnancy-related fluid retention causes the kidneys to enlarge and frequently results in physiologic hydronephrosis [6].

**Figure 1:**
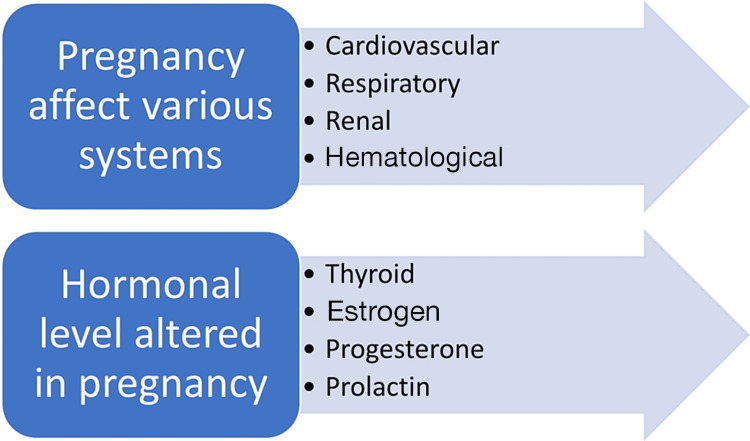
Basic outline of the affected systems and the hormones altered in pregnancy Image credit: Sohan Jee

Below is a basic outline of the affected systems and the hormones altered in pregnancy (Figure 1).

## Review

Search methodology

The review article focuses on the physiological changes brought on by hormonal changes in pregnant women and how these affect kidney function. Comprehensive literature searches were conducted using electronic databases like PubMed, Google Scholar, and ScienceDirect as part of the search technique. Keywords like “physiological changes,” “pregnancy hormones,” “renal function,” and “kidney” were employed to find pertinent papers published from the beginning to the most recent date. Focusing on hormonal changes during pregnancy and their impact on renal physiology, studies with varied populations and experimental designs were considered. The article synthesizes information from several papers to provide a thorough understanding of the complex interactions between hormonal changes and kidney function in pregnant women.

**Figure 2:**
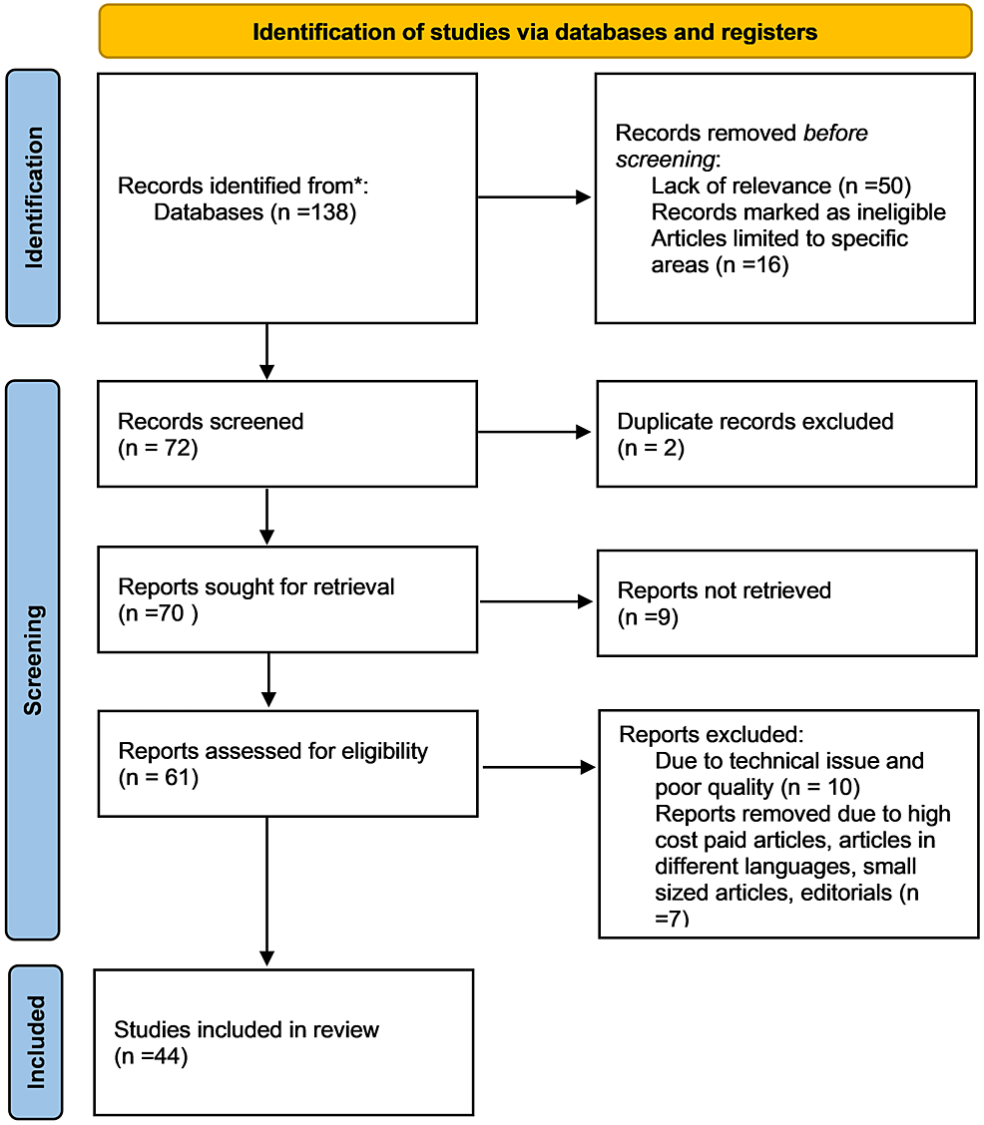
PRISMA flow diagram depicts search processes on the topic “Physiological Changes in Pregnant Women Due to Hormonal Changes”

The PRISMA flow diagram below shows the mathematical algorithm for the number of articles and references used in this study (Figure 2).

Thyroid

The thyroid gland grows more prominent and suffers from follicular hyperplasia during pregnancy. TSH receptors can be activated by beta-human chorionic gonadotropin [7]. At the 20th week of pregnancy, the amount of thyroxine-binding globulin is two to three times higher than before pregnancy due to increased liver production and decreased estrogen clearance. As a result, women who are using supplements during early pregnancy may need to increase their thyroxine dosage. The thyroid function of pregnant women who have been diagnosed with hypothyroidism should be checked as soon as is practical [8]. Large biotin intake may produce a Graves’ disease-like picture with high fT4, elevated fT3, and enhanced TSH receptor antibodies due to immunoassay interference. Thyroid hormone is measured during amniocentesis; the mother may create thyroid hormone antibodies in response to fetal tissue epitopes, which could result in an abnormal increase in fT4 and fT3 [9].

The above information is depicted in tabular form in Table 1.

**Table 1:** Thyroid level changes during pregnancy TSH, thyroid-stimulating hormone Table credit: Sohan Jee

Topic	Information
Thyroid changes during pregnancy	The thyroid gland enlarges and experiences follicular hyperplasia during pregnancy. The level of T3-binding globulin rises between two and three times by the 20th week due to increased liver production and decreased estrogen clearance.
TSH receptor activation	TSH receptors can be activated by beta-human chorionic gonadotropin.
Monitoring hypothyroidism in pregnant women	Pregnant women diagnosed with hypothyroidism should have their thyroid function checked as soon as practical.
Thyrotoxicosis causes during pregnancy	Graves’ disease is a common cause of thyrotoxicosis during pregnancy.
Biotin interference and Graves’ disease-like symptoms	Due to immunoassay interference, a high biotin intake can mimic Graves’ illness with raised fT4, fT3, and heightened TSH receptor antibodies.
Thyroid hormone antibodies in pregnancy	Thyroid hormone antibodies may be produced by the mother in response to fetal tissue epitopes, potentially causing abnormal increases in fT4 and fT3.
Thyroid hormone measurement during amniocentesis	Thyroid hormone levels can be measured during amniocentesis, but caution is needed due to potential interference from antibodies created by the mother in response to fetal tissue epitopes.

Calcium/phosphorus-related level

During pregnancy, there is a slight decrease in calcium, magnesium, and phosphate levels. Due to an average decline in albumin levels, the corrected calcium findings may appear higher than they are. Measuring ionized calcium is advisable since its levels remain constant throughout pregnancy. Parathyroid hormone (PTH) levels fall by about 50% in the second and third trimesters, while PTH-related peptide (PTHrP) concentrations increase. The primary hormone controlling calcium homeostasis is PTHrP, primarily placental, and peaks in the third trimester of pregnancy [10]. From the 12th week of pregnancy, urinary calcium excretion escalates two to three times due to heightened calcium absorption and GFR. This rise in urinary excretion is due to increased GFR and calcium absorption; calcium excretion increases by two to three times. Studies have shown that in genetically identified familial hypocalciuric hypercalcemia cases during pregnancy, urinary calcium concentration reached as high as 8.6 mmol/L, the urinary calcium/creatinine ratio of clearance was 0.02, calcium concentration grew to 8.6 mmol/L, and calcium correction increased to 3.35 mmol/L [11].

Additionally, in the third trimester, serum magnesium levels experienced a 30% reduction compared to pre-conception levels due to a decline in serum magnesium. According to nuclear magnetic resonance spectroscopy measurements, ionized, RBC, and intracellular free magnesium concentrations in the brain and muscle are lower in pregnancy than in non-pregnant controls. Approximately 25% more magnesium is excreted in the urine during pregnancy [12].

Respiratory

Maternal respiratory physiology changes due to hormonal and biochemical changes in the central respiratory center, regional changes in respiratory smooth muscle, or mechanical changes in the growing fetus. The respiratory center is stimulated by progesterone in the blood, increasing minute ventilation, mainly by increasing tidal volume (by about 40%) and respiratory rate (by about 15%) [7]. To accommodate the developing fetus’s increased oxygen requirements, the respiratory system undergoes several physiological and anatomical changes, including increases in tidal volume, ventilation, and respiratory rate. Functional residual capacity, total lung capacity, and expiratory reserve volume decline during pregnancy. This is mainly because increased progesterone levels have affected the flexibility of the ligaments. Additionally, a contributing factor to these harmful effects on the respiratory system is the increase in intra-abdominal pressure that occurs as the uterus grows [13].

Renin-angiotensin-aldosterone system

Compared to non-pregnant levels, plasma aldosterone concentrations rise roughly three to eight times, plateauing in the third trimester [14]. Similar to this, term pregnancy has approximately eight-fold higher 24-hour urine aldosterone excretion than pre-pregnancy. By eight weeks of gestation, plasma renin activity has increased four-fold and seven-fold by term. Aldosterone-to-renin ratios may decrease, which could affect the diagnosis of primary aldosteronism. Because of worries concerning salt and volume in the loading process, dynamic tests to identify primary aldosteronism are generally avoided during pregnancy. However, results are reported following a saline infusion for healthy pregnant women [15].

Pregnancies without a corpus luteum did not increase the amount of circulating prorenin. The prorenin rise in these pregnancies was comparable to the renin rise in modesty, and prorenin decreased concurrently with renin after delivery. Recent data from 277 women with no corpus luteum, one corpus luteum, or more than one corpus luteum further supported the link between prorenin levels and corpus luteum number. Together, these findings show that the ovaries primarily produce prorenin levels in pregnant women, whereas the kidneys drive renin upregulation in pregnancy [16].

Cardiovascular

Early in pregnancy is when most cardiovascular changes take place. Increased levels of progesterone, estrogen, and prostaglandins in the bloodstream cause vascular smooth muscle to relax, which lowers pulmonary and systemic vascular resistance. Cardiac output gradually rises during the third trimester, eventually rising by up to 30-50%. The increase in cardiac output is brought on by ventricular hypertrophy, which also causes an increase in heart rate and stroke volume [17]. Pre-eclampsia (PET) is a pregnancy-related condition characterized by high blood pressure (hypertension) and damage to other organs, typically the liver and kidneys [12,17]. PET usually occurs after 20 weeks of gestation and can lead to complications for both the mother and the baby if left untreated [13,18]. When a woman has severe pulmonary swelling and PET abnormalities, it is essential to consider the possibility of myocardial injury in diabetic pregnancies, even though cardiac troponin I levels are unaffected in healthy pregnancies in as many as 25% of PET-complicated conceptions. Elevated levels of brain natriuretic peptide are linked to chronic hypertension, gestational hypertension, and PET, and these associations may not go away until three to six months after childbirth [18]. During pregnancy, coronary angiography can be done without risk.

Sex hormone

Elevated blood levels of reproductive hormones, such as estrogen and progesterone, contribute to many of the physiological changes related to pregnancy. Additionally, the placenta secretes hormones that impact several bodily systems, including relaxin, human placental lactogen, and human chorionic gonadotropin [19]. Estradiol levels rise progressively throughout pregnancy, reaching 100 times prenatal levels by the third trimester. The levels of progesterone and 17-hydroxyprogesterone are also growing steadily and clearly. Pre-pregnancy testosterone level elevations are noted multiplied by five. During pregnancy, the levels of sex hormone-binding globulin increase by about five times. Neo-angiogenesis, the formation of tissues that support lactation and eventually develop into the placenta, is sparked by elevated estrogen levels. These endocrine alterations are what cause the common pregnant woman’s symptoms of weakness, vomiting, bowel movements, and headache [20].

Because immune cells have hormone receptors, sex hormones like estrogen, progesterone, androgens, and prolactin can affect several parts of the immune system’s function and may affect MS risk, activity, and development. The kind of target cell, the receptor subtype expressed on that cell type, and the concentration of sex hormones will all affect the effects of the hormones differently. Therefore, it is important to clarify the intricate relationships between sex hormones, sex chromosomes, and immune response genes to comprehend how gender influences MS and autoimmunity in general [3]. The impact of sex hormones on immune cells is described in Table 2.

**Table 2:** Impact of sex hormones on immune cells

Sex hormone	Target cell	Receptor subtype	Effect on immune function
Estrogen (estradiol)	Macrophages	Estrogen receptor alpha and estrogen receptor beta	Increases antibody production and reduces inflammation
Progesterone	T cells and B cells	Progesterone receptor	Suppresses inflammation and modulates immune response
Androgens (testosterone)	T cells, B cells, and macrophages	Androgen receptor	Suppresses immune response and reduces inflammation
Prolactin	Macrophages and dendritic cells	Prolactin receptor	Modulates inflammation and affects antigen presentation

Renal

The increase in cardiac output was accompanied by a 50% increase in renal blood flow and GFR. There may be a 40% reduction in serum urea and creatinine from pre-pregnancy levels. Urinary protein and glucose levels increase as renal absorption of these molecules (as well as other molecules like bicarbonate and other electrolytes) is surpassed by glomerular filtration [7]. Progesterone can increase RPF and GFR, but it cannot explain the extent of the increase seen during pregnancy. The placenta, decidua, and corpus luteum release the vasodilator hormone relaxin. It is associated with renal physiology during pregnancy in rodents through increased vascular gelatinase activity acting through the endothelial endothelin B receptor-nitric oxide pathway [21]. The tremendous pressure that the developing fetus puts on the mother causes the mother’s bladder capacity to decrease, leading to frequent urination, one of the most common pregnancy symptoms. In addition, an increase in renal blood flow can lead to a 50% increase in GFR and a decrease in serum creatinine levels [22].

In a healthy pregnancy, proteinuria physically doubles in intensity. Even when PET is absent, proteinuria typically doubles during pregnancy in people with underlying proteinuria. Diabetes and lupus nephropathy are the two primary exceptions [1]. With the increased proteinuria brought on by diabetic nephropathy and the average increase, it could be more challenging to recognize superimposed positron emission tomography during the third trimester due to changes in blood pressure. The ratio of soluble FMS-like tyrosine kinase 1 to placental growth factor, which is substantially larger in PET than in females with chronic kidney disease, may be used to diagnose PET in females with diabetic nephropathy [23].

Hematological

Because of the physiological strain experienced during pregnancy, there is an elevation in WBC counts, especially in neutrophils, during the initial trimester. The probable reason for this is the diminished ability of neutrophils to undergo apoptosis during pregnancy, likely due to an upsurge in inhibitory factors present in the serum. The quantity of myelocytes and metamyelocytes also rises, suggesting increased erythrocytes and higher marrow activity throughout pregnancy. Additionally, the lymphocyte count decreases in the first and second trimesters while increasing in the third [24]. Additionally, studies have shown that platelet counts decrease during pregnancy, particularly in the third trimester. This condition, known as gestational thrombocytopenia, is brought on by the hemodilution that occurs during pregnancy, along with a rise in platelet activation and clearance. Due to the rise in estrogen levels during pregnancy, coagulation factor levels are also increased, which causes a prothrombotic state, especially in the third trimester [25]. However, venous thromboembolism, one of the leading causes of maternal death, is becoming more prevalent in pregnant women both throughout pregnancy and after giving birth [26-29]. The preferred medication for treating venous thromboembolism in pregnancy is low-molecular-weight heparin. The majority of women use anticoagulants twice daily throughout pregnancy or after delivery. However, population pharmacokinetic research demonstrated the effectiveness of a one-day dose, adjusted for weight, in treating venous thromboembolism [30]. The hematological system undergoes widespread adaptations during the peripartum period, and risks for anemia, thromboembolism, and consumptive coagulopathies significantly rise. Increased aldosterone secretion (by renin-angiotensin axis activation) increases the volume of plasma and the overall water content of the body as a result. Erythropoiesis also increases by approximately 30% [7].

Gastrointestinal

Heartburn during pregnancy is typical. This is explained by a drop in the pH of gastric secretions, an increase in the volume of secretions, and a drop in the tone of the lower esophageal sphincter [31]. Progesterone activity on smooth muscle cells causes a decrease in lower esophageal sphincter tone, which causes heartburn symptoms and contributes to nausea and vomiting [32]. Around 80% of pregnant individuals experience nausea and vomiting, although the severity and presentation might vary [33,34]. It can start as early as the second week, continue through the second trimester, and, in some circumstances, until 37 weeks of pregnancy or full term [35-37]. There is debate regarding whether pregnancy affects stomach emptying and motility and whether this affects the peak blood concentration of oral medications. According to some research, the delay is brought on by elevated levels of progesterone and estrogen, which cause the smooth muscle cells of the digestive tract to relax [38,39]. In addition to its involvement in enhancing nausea and vomiting during pregnancy, this drop can frequently cause constipation, bloating, and discomfort in patients. This decrease may also delay the system’s peak concentration of oral medications.

In contrast to studies demonstrating no delay in motility throughout pregnancy [40,41], some investigations have found delayed stomach emptying, particularly early in pregnancy [40,42]. A delay in stomach emptying during pregnancy might impact the absorption of some drugs, including acetaminophen. Additionally, both pregnant patients and non-pregnant patients may experience altered absorption profiles from the concurrent use of various drugs [43]. For instance, patients are advised not to take proton pump inhibitors and iron supplements together since they may reduce levothyroxine absorption [44].

## Conclusions

To sum up, the process of pregnancy involves a great physiological change that involves subtle adjustments to a number of organ systems and is controlled by a complex interplay of hormone variations. It is essential to comprehend these changes in order to evaluate endocrine abnormalities during this crucial time. Significant changes occur in the endocrine system, particularly in the thyroid and its feedback mechanisms. For an appropriate assessment, elements like biotin and potential interference from factors like enlarged thyroid glands and elevated thyroxine-binding globulin must be carefully taken into account. Pregnancy also has a substantial impact on calcium and phosphorus levels, which affect PTH and make it difficult to diagnose linked disorders. Hormonal factors regulate respiratory adaptations, which change breathing to accommodate the developing fetus’s changing metabolic needs. A rise in cardiac output and vascular relaxation caused by hormone surges are examples of cardiovascular alterations.

Significant changes to the renin-angiotensin-aldosterone system affect fluid balance and may change diagnostic criteria. Importantly, sex hormones have a significant impact on immune responses, which affect the occurrence and course of disease, particularly in disorders like MS. To meet the needs of pregnancy, the kidneys in the renal domain exhibit increased filtration rates and altered electrolyte management. To fulfill heightened needs and reduce potential dangers, the hematological system also goes through continuous modifications. These physiological adjustments, which are essential for a healthy pregnancy, highlight the demand for specially designed medical care for expectant women. For optimal mother and fetal health, a thorough understanding of these complex shifts is necessary, as it will guide safe diagnostic and treatment approaches throughout this transformational stage. To improve prenatal treatment and extend our understanding of endocrine diseases in pregnancy, future research should go deeper into figuring out the complexity of these physiological changes. To some extent, the gastrointestinal tract is also affected.
